# Arrhythmogenic mechanisms in the isolated perfused hypokalaemic murine heart

**DOI:** 10.1111/j.1748-1716.2006.01643.x

**Published:** 2007-01-01

**Authors:** M J Killeen, G Thomas, I S Gurung, C A Goddard, J A Fraser, M P Mahaut-Smith, W H Colledge, A A Grace, C L-H Huang

**Affiliations:** 1 Physiological Laboratory, University of Cambridge Cambridge, UK; 2 Section of Cardiovascular Biology, Department of Biochemistry, University of Cambridge Cambridge, UK

**Keywords:** arrhythmogenesis, hypokalaemia, mouse heart

## Abstract

**Aim:**

Hypokalaemia is associated with a lethal form of ventricular tachycardia (VT), torsade de pointes, through pathophysiological mechanisms requiring clarification.

**Methods:**

Left ventricular endocardial and epicardial monophasic action potentials were compared in isolated mouse hearts paced from the right ventricular epicardium perfused with hypokalaemic (3 and 4 mm [K^+^]_o_) solutions. Corresponding K^+^ currents were compared in whole-cell patch-clamped epicardial and endocardial myocytes.

**Results:**

Hypokalaemia prolonged *epicardial* action potential durations (APD) from mean APD_90_s of 37.2 ± 1.7 ms (*n* = 7) to 58.4 ± 4.1 ms (*n* =7) and 66.7 ± 2.1 ms (*n* = 11) at 5.2, 4 and 3 mm [K^+^]_o_ respectively. *Endocardial* APD_90_s correspondingly increased from 51.6 ± 1.9 ms (*n* = 7) to 62.8 ± 2.8 ms (*n* = 7) and 62.9 ± 5.9 ms (*n* = 11) giving reductions in endocardial–epicardial differences, ΔAPD_90_, from 14.4 ± 2.6 to 4.4 ± 5.0 and −3.4 ± 6.0 ms respectively. Early afterdepolarizations (EADs) occurred in epicardia in three of seven spontaneously beating hearts at 4 mm [K^+^]_o_ with triggered beats followed by episodes of non-sustained VT in nine of 11 preparations at 3 mm. Programmed electrical stimulation *never* induced arrhythmic events in preparations perfused with normokalemic solutions yet induced VT in two of seven and nine of 11 preparations at 4 and 3 mm [K^+^]_o_ respectively. Early outward K^+^ current correspondingly fell from 73.46 ± 8.45 to 61.16±6.14 pA/pF in isolated *epicardial* but not *endocardial* myocytes (*n* = 9) (3 mm [K^+^]_o_).

**Conclusions:**

Hypokalaemic mouse hearts recapitulate the clinical arrhythmogenic phenotype, demonstrating EADs and triggered beats that might *initiate* VT on the one hand and reduced transmural dispersion of repolarization reflected in ΔAPD_90_ suggesting *arrhythmogenic substrate* on the other.

Cardiac K^+^ channels govern a range of important physiological functions that include heart rate and action potential (AP) waveform and duration (Tamargo *et al.* 2004). In particular, cardiac AP repolarization is regulated by a variety of K^+^ channel currents that include the transient outward current, *I*_to_, the rapidly activating delayed rectifier current, *I*_Kr_, and the inwardly rectifying current, *I*_K1_. Reductions in outward K^+^ channel currents have been associated with impaired repolarization with a consequent increase in AP duration and a prolonged surface electrocardiographic QT interval (Choy *et al.* 1997).

Both increases and decreases in extracellular K^+^ ([K^+^]_o_) have been associated with potentially life-threatening arrhythmias (Curtis *et al.* 1993). At the cellular level, low [K^+^]_o_ has been shown to reduce K^+^ currents and enhance the potency of agents that block K^+^ channels (Sanguinetti & Jurkiewicz 1992, Yang & Roden 1996). Hypokalaemia is a recognized risk factor alongside bradycardia for the development of torsade de pointes (TdP), a life-threatening form of ventricular tachycardia (VT), in which the QRS complexes appear to twist about the isoelectric line (Antzelevitch *et al*. 1996, Berthet *et al.* 1999, He & MacGregor 2001). Currently two theories, not necessarily exclusive, preside over the induction of TdP: (1) delayed repolarization, resulting from AP prolongation leads to early afterdepolarizations (EADs) that interrupt the otherwise smooth repolarization phase of the AP, and may give rise to salvos of premature triggered beats and TdP (Roden 2004). (2) Heterogeneous distribution of cardiac ion channel currents through the thickness of the ventricular wall creates a transmural dispersion of repolarization (TDR), which may exacerbate upon AP lengthening (Papadatos *et al.* 2002). Ordinarily, a TDR, and therefore a transmural gradient in refractoriness, plays an important role in the spread of repolarization throughout the ventricle (i.e. proceeding from the epicardium to the endocardium). Agents that affect action potential duration (APD) to differing extents across the ventricular wall, would result in altered APD transmural gradients, and hence refractoriness, both of which are potentially arrhythmogenic mechanisms (Janse & Wit 1989).

The multiple risk factor intervention trial (Cohen *et al.* 1987) reported a 28% increase in ventricular arrhythmias for every 1 mm reduction in serum K^+^ amongst male hypertensive patients receiving diuretic therapy. Furthermore, corrections of serum K^+^ through intravenous or oral potassium administration have been reported to reduce the QT interval in long QT (LQT) patients, and may thus help prevent subsequent sudden cardiac death (SCD) (Choy *et al.* 1997, Etheridge *et al*. 2003).

The Nernst equation predicts that a reduction in [K^+^]_o_ should increase the driving force for outward current through K^+^ channels and therefore increase *I*_K_. However, studies of *I*_to_, *I*_Kr_ and *I*_K1_ in human atrial myocytes, guinea-pig myocytes and sheep cardiac purkinje fibres, respectively, have demonstrated that reduced [K^+^]_o_ actually decreases these K^+^ currents (Carmeliet 1982, Sanguinetti & Jurkiewicz 1992, Firek & Giles 1995). These findings could help explain the cardiac AP prolongation that has been observed at low [K^+^]_o_, a well-recognized clinical phenomenon, that may play an important role in the genesis of arrhythmias such as TdP (Ginant *et al*. 1991; Yang & Roden 1996).

A reduction in [K^+^]_o_ is a common experimental manoeuvre employed in isolated cardiac tissue and whole-heart preparations when assessing the arrhythmic tendency of drugs implicated in acquired long QT syndrome (LQTS) or establishing indirect, pharmacological models of LQTS, or in assessing the pathogenesis of cardiac arrhythmias (Eckardt *et al.* 1998, Milberg *et al.* 2002, 2005). These studies have lowered [K^+^]_o_ in combination with the administration of a wide range of compounds thought to be implicated in the development of TdP. Furthermore, in many of these studies, it was actually necessary to reduce [K^+^]_o_ to induce arrhythmias, even in the presence of known arrhythmogenic agents (Milberg *et al.* 2005). This suggests that [K^+^]_o_ is an important trigger for cardiac arrhythmias in its own right, yet such reports did not themselves assess the effects that reductions in [K^+^]_o_*by itself* may have upon the arrhythmic tendency of these cardiac preparations.

Despite studies documenting the effects of hypokalaemia upon *I*_to_, *I*_Kr_ and *I*_K1_ in isolated cardiac myoctes and tissue preparations and the established clinical association of hypokalaemia and TdP (Berthet *et al.* 1999), the existence of such a precise link has not yet been proven. Studies in the intact isolated heart have the advantage of containing all myocardial cell types whilst maintaining intercellular coupling, and could thus provide more physiologically relevant information regarding the induction and propagation of cardiac arrhythmia. The purpose of this study, therefore, was to determine the intrinsic arrhythmogenic effects of hypokalaemia in the isolated, Langendorff-perfused murine whole-heart model, and to assess if an increased arrhythmic state is accompanied by an altered transmural gradient of APD.

## Methods

### Experimental animals

The mice used in this study were kept in an animal house at room temperature and subjected to a consistent 12 h : 12 h light : dark cycle and fed with sterile rodent chow, having access to water at all times. Wild-type (WT) 129 background male and female mice aged 5–7 months were used in all experiments.

### Langendorff-perfused preparation

The experiments used a Langendorff-perfused preparation that has been previously adapted for murine hearts (Balasubramaniam *et al.* 2003). Briefly, mice were killed by cervical dislocation in accordance with Schedule 1 of the UK Animals (Scientific Procedures) Act 1986. The heart was then quickly excised and submerged in ice-cold bicarbonate-buffered Krebs–Henseleit solution containing in mm: 119 NaCl, 25 NaHCO_3_, 4 KCl, 1.2 KH_2_PO_4_, 1 MgCl_2_, 1.8 CaCl_2_, 10 glucose and 2 sodium pyruvate. The solution was bubbled with a 95% O_2_–5% CO_2_ mixture (British Oxygen Company, Manchester, UK). The aorta was cannulated under the buffer surface using a 21-gauge custom-made cannula, and was attached to the cannula needle using a micro-aneurysm clip (Harvard Apparatus, Edenbridge, UK). The preparation was then transferred to the perfusion apparatus, to which the cannula was attached, and perfusion commenced in a retrograde manner via the aorta with the abovementioned bicarbonate-buffered Krebs–Henseleit solution. Before entering the aorta, buffer was passed through 200 and 5 *μ*m filters (Milipore, Watford, UK) and warmed to 37 °C by means of a water jacket and circulator (Model C-85A, Techne, Cambridge, UK). Perfusion was maintained at a constant flow rate of 2–2.5 mL min^−1^ using a peristaltic pump (Watson–Marlow Bredel pumps model 505S, Falmouth, Cornwall, UK). Following the start of perfusion, healthy, experimentally viable hearts regained a pink colouration and spontaneous rhythmic contraction with warming. In 10% of experiments, hearts were discarded because of signs of ischaemia after cannulation and perfusion.

### Perfused heart electrophysiological measurements

In the present experiments, a paired (1-mm inter-pole spacing) platinum stimulating electrode was placed on the basal surface of the right ventricular epicardium. Prior to experimental procedures, hearts were paced for 10 min at 8 Hz using 2-ms square-wave stimuli with amplitudes set to three times the excitation threshold (Grass S48 stimulator, Grass-Telefactor, Slough, UK).

Epicardial MAP recordings were obtained using a MAP electrode (Linton Instruments, Harvard Apparatus, UK) placed on the basal surface of the left ventricular epicardium. The epicardial MAP electrode was gradually positioned until a gentle but stable contact pressure was achieved. This resulted in a recording of MAP signals. For endocardial recordings, a small access window was created in the interventricular septum to gain access to left ventricular endocardium (Casimiro *et al.* 2001). A custom-made endocardial MAP electrode constructed from two twisted strands of Teflon-coated (0.25 mm diameter) silver wire (99.99% purity) (Advent Research Materials Ltd, Oxford, UK) that had been previously galvanically chlorided to eliminate DC offset, was positioned on to the left ventricular free wall under a stable contact pressure until MAP signals were achieved. MAPs were amplified, band-pass filtered (0.5 Hz to 1 kHz: Gould 2400S, Gould-Nicolet Technologies, Ilford, Essex, UK) and digitized (1401 plus MKII, Cambridge Electronic Design, Cambridge, UK). MAPs were extracted and analysed (spike ii version 4: Cambridge Electronic Design) to derive the precise duration of the digitized signals. The recordings were deemed reproducible and, hence of an acceptable standard for analysis if they had the following properties: a stable baseline, a rapid upstroke phase with consistent amplitude, a smooth contoured repolarization phase and a stable duration [MAP duration at 90% repolarization (APD_90_) was reproducible within 2 ms under baseline conditions].

### Experimental protocol

A standard pacing protocol (basic cycle length, BCL of 125 ms) that corresponded to physiological whole-animal heart rates (Papadatos *et al.* 2002) was initiated for periods of up to 20 min to measure APD at 50%, 70% and 90% repolarization. External pacing stimuli were subsequently withdrawn from all preparations, leading to a significantly reduced, intrinsic heart rate corresponding to a BCL of approximately 400 ms. Reduced heart rates are a known risk factor for the development of repolarization abnormalities such as EADs and triggered beats that may underlie the induction of VT (Roden & Hoffman 1985). Epicardial MAPs were recorded for periods of up to 20 min from isolated, perfused WT mouse hearts under intrinsic pacing conditions. Following this, programmed electrical stimulation (PES) of the heart was carried out using an adaptation of the corresponding clinical techniques (Saumarez & Grace 2000, Balasubramaniam *et al.* 2003). PES procedures began by applying standard pacing stimuli at a BCL of 125 ms for 25 s. Following this, a drive train of eight-paced beats (S1) again at a BCL of 125 ms preceded an extrastimulus (S2) every ninth beat. S1S2 intervals initially equalled the pacing interval and then were progressively reduced by 1 ms with each nine-beat cycle until ventricular refractoriness was reached, at which point the S2 stimulus elicited no MAP. BCL pacing protocols of 125 ms, corresponding to physiological whole-animal heart rates (Papadatos *et al.* 2002), were used in all paced experiments. Recordings were subsequently repeated following a 20-min wash-in of a reduced [K^+^]_o_ perfusate, of either 4 or 3 mm.

We used two methods to quantify changes in transmural gradients of repolarization. Firstly, ΔAPD_90_ was calculated from the difference between the mean endocardial and epicardial APD_90_ values, giving positive results where the endocardial value exceeded the epicardial value, and negative results where the epicardial value was greater. Secondly, TDR was defined as the positive part of the ΔAPD_90_ as described on earlier occasions (Kirchhof *et al.* 1996). An EAD was defined as a positive deflection that interrupted the smooth repolarization phase of the AP. A triggered beat was similarly described as a positive deflection in the smooth repolarization phase of the AP whose amplitude approximately matched the amplitude of the initial AP. Arrhythmias were defined as a ventricular tachyarrhythmia of more than five-cycle duration that were typically self-terminating. Following cannulation and subsequent perfusion of hearts, approximately 10% of preparations were discarded because of signs of ischaemia.

### Isolation of single-ventricular myocytes

Epicardial and endocardial myocytes were dissociated enzymatically from the left ventricle. Following cannulation, the heart was perfused in a retrograde fashion with Krebs–Henseleit buffer, warmed to 37 °C by means of a water jacket and circulator (Techne model C-85A), at a rate of 2–2.5 mL/min for 5 min, until the heart regained a homogenous pink colouration and began contracting spontaneously. The heart was then perfused for 5 min with a nitrilotriacetic acid-based perfusion buffer containing (in mm): 125 NaCl, 4.75 KCl, 5 MgSO_4_, 10 HEPES, 5 sodium pyruvate, 20 glucose, 20 taurine and 4.5 nitrilotriacetic acid. Following this, the heart was perfused with a digestion buffer for 12–15 min containing (in mm): 125 NaCl, 4.75 KCl, 5 MgSO_4_, 10 HEPES, 5 sodium pyruvate, 20 glucose, 20 taurine, 0.6 CaCl_2_ and 1 mg/mL collagenase type 2 (Worthington, UK), 1 mg/mL hyaluronidase (Sigma, Poole, UK). After this period, a small pair of 90-degree curved forceps was used to tear off a thin layer of left ventricular epicardial tissue. After epicardial tissue had been harvested, a surgical blade was used to create an incision along the length of the heart, to gain access to the left ventricular endocardial surface. As before, a pair of forceps was used to tear off thin sections of endocardial tissue. Epicardial and endocardial tissue samples were placed in separate tubes containing digestion buffer in addition to 1 mg/mL bovine serum albumin (Sigma) for 5 min before gentle trituration for a further 5 min in the same solution. Tissue samples were subsequently spun down in a centrifuge machine (1000 rpm for 3 min) before the supernatant from the epicardial and endocardial tissue tubes was discarded and replaced with a wash buffer containing (in mm): 135 NaCl, 1.1 MgCl_2_, 1.8 CaCl_2_, 5.4 KCl, 10 Hepes, 10 Glucose and pH was adjusted to 7.35 with NaOH. Epicardial and endocardial myocytes were stored in the aforementioned wash buffer and were studied within 4–6 h. Following initial perfusion of the heart, all subsequent steps were performed at room temperature.

### Single-cell electrophysiology

Conventional whole-cell patch-clamp recording in voltage clamp mode were carried out using an Axopatch 200B amplifier (Axon Instruments, CA, USA) coupled to a Digidata series computer interface and controlled by pClamp software (Axon Instruments). Pipettes (1–4 MΩ) were pulled from borosilicate glass capillaries (1.5 mm outer and 0.86 inner diameter, GC150–10; Harvard Apparatus Ltd). Extracellular buffer contained (in mm): 135 NaCl, 1.1 MgCl_2_, 1.8 CaCl_2_, 5.4 KCl, 10 Hepes, 10 Glucose and pH was adjusted to 7.35 with NaOH. Intracellular pipette saline contained (in mm): 130 KCl, 1 MgCl_2_, 10 Hepes, 5 Mg-ATP, 5 Na_2_-creatine phosphate and pH was adjusted to 7.2 with KOH. After formation of gigaseal, whole-cell configuration was achieved by applying gentle suction through pipette and ZAP. Up to 75% series resistance compensation was achieved. Transient outward potassium currents and inward currents were triggered by applying a series of 10 mV incremental voltage pulses from −100 to 50 mV from a holding potential of −60 mV.

### Data analysis and statistics

Single-cell electrophysiological data and whole-heart MAP data were initially imported into Microsoft excel. All data are expressed as means ± SEM. For whole-heart data, comparisons were made using anova (SPSS software) and for single-cell electrophysiological data comparisons were made using Student's *t*-test, with values of *P* < 0.05 being considered significant.

## Results

Hypokalaemia is a known risk factor for the development of a lethal form of VT termed torsade de pointes, although the underlying physiological mechanisms responsible for this remain unclear (Roden *et al*. 1996). The experiments sought to investigate the intrinsic arrhythmogenicity induced by hypokalaemia by recording left ventricular epicardial and endocardial monophasic action potentials (MAPs) from isolated, perfused mouse hearts, and to determine whether arrhythmogenicity was associated with the occurrence of repolarization abnormalities such as EADs and triggered beats, an altered transmural gradient of repolarization or a combination of the two.

### Stability of endocardial and epicardial MAP recordings

Experimental data were initially obtained from recordings of MAPs from isolated, perfused WT mouse hearts under normokalaemic conditions (5.2 mm [K^+^]_o_) to establish the control phenotype. The procedures were then repeated following reductions in [K^+^]_o_. Following cannulation and perfusion of the murine hearts, the electrophysiological parameters of MAP waveform morphology, amplitude and duration reached a steady state within 10 min. Following this stabilization period, MAP recordings and pacing thresholds remained highly reproducible throughout the experimental protocol.

**Figure 1:**
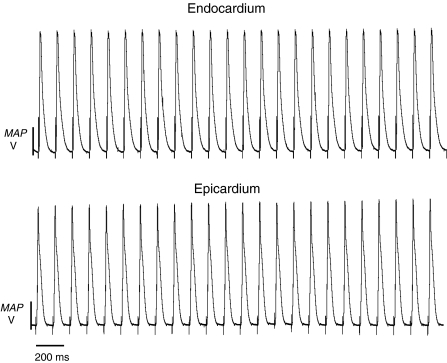
Representative example of left ventricular endocardial and epicardial MAP recordings under control, normokalaemic conditions of 5.2 mm [K^+^]_o_ in an isolated Langendorff-perfused WT mouse heart. Under control conditions, MAP waveform characteristics remained highly stable and reproducible throughout all recordings.

The MAPs recorded fulfilled the previously documented murine cardiac electrophysiological criteria in possessing a triangular morphology, a rapid upstroke phase, a smooth repolarization phase, and closely resembled murine ventricular MAPs from earlier studies (Guo *et al.* 1999) (Fig. 1). No MAP waveform repolarization abnormalities in either the epicardium or endocardium were ever seen under normokalemic (5.2 mm [K^+^]_o_) conditions. Both epicardial and endocardial MAP amplitudes and durations remained highly stable throughout the duration of experimental recording procedures, with APD_90_ values being reproducible within 2 ms under normokalemic conditions (Tables 1 & 2) in 25 separate preparations, further validating this experimental set-up.

**Table 1:** Epicardial action potential durations (APDs) under varying [K^+^]_o_ (mm) conditions

Parameter (ms)	5.2 (*n* = 7)	4 (*n* = 7)	3 (*n* = 11)
APD_50_	7.8 ± 0.8	16.9 ± 3.6*	19.5 ± 2.4*
APD_70_	19.7 ± 2.3	31.4 ± 4.9	37.2 ± 2.9*
APD_90_	37.2 ± 1.7	58.4 ± 4.1*	66.7 ± 2.1*

* *P* < 0.05 vs. baseline.

**Table 2:** Endocardial action potential durations (APDs) under varying [K^+^]_o_ (mm) conditions

Parameter (ms)	5.2 (*n* = 7)	4 (*n* = 7)	3 (*n* = 11)
APD_50_	19.7 ± 1.3	19.7 ± 0.8	17.2 ± 1.6
APD_70_	32.2 ± 1.6	32.7 ± 3.1	34.7 ± 2.4
APD_90_	51.6 ± 1.9	62.8 ± 2.8*	62.9 ± 5.9*

* *P* < 0.05 vs. baseline.

### Hypokalaemia modifies the regional heterogeneity of murine ventricular repolarization

The experiments then proceeded to investigate whether reductions in [K^+^]_o_ affected the transmural gradient of repolarization in the intact, isolated, perfused mouse heart. Local activation time is the time measured from the point of electrical stimulus to the maximal amplitude of the AP repolarization time is obtained by the addition of local activation times to MAP duration; however, in the present study we only observed insignificant changes in local activation time in the presence of reduced [K^+^]_o_ (data not shown). This finding is in keeping with a previous study in which perfusion of isolated rabbit hearts with amiodarone led to no significant increase or decrease in local activation times (Kirchhof *et al.* 2003). With this in mind, the present experiments measured changes in the transmural gradient of repolarization by first calculating a ΔAPD_90_ from the difference between the epicardial APD_90_ and the endocardial APD_90_; this gave a positive value where the endocardial APD_90_ exceeded the epicardial APD_90_, and a negative value if the opposite was the case. However, TDR was then obtained from the positive part of this ΔAPD_90_ as defined on earlier occasions (Kirchhof *et al.* 1996).

**Figure 2:**
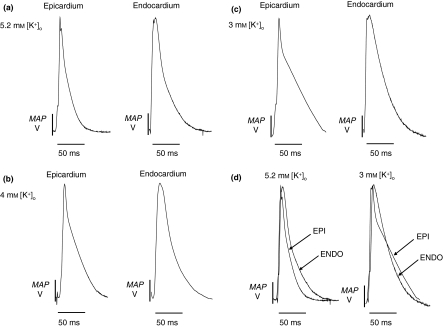
Representative MAP recordings from the left ventricular endocardium and epicardium of isolated, Langendorff-perfused WT mouse hearts during a standard pacing protocol at a basic cycle length of 125 ms under (a) control conditions and following perfusion with hypokalaemic solutions of 4 mm (b) and 3 mm [K^+^]_o_ (c). Perfusion with 4 and 3 mm [K^+^]_o_ leads to marked prolongation of both endocardial and epicardial APD. (d) Overlaid epicardial and endocardial traces shown in panels (a) and (c).

**Figure 3:**
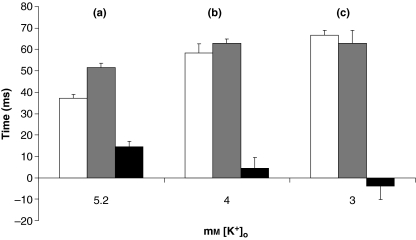
Steady-state epicardial and endocardial APD measured at 90% repolarization (APD_90_), and ΔAPD_90_ values (white, grey and black columns respectively) under (a) control conditions (seven hearts), and following perfusion with hypokalaemic solutions (b) 4 mm (seven hearts) and (c) 3 mm [K^+^]_o_.

Fig. 2 shows representative epicardial and endocardial MAPs recorded from isolated, mouse hearts perfused with either normokalemic (5.2 mm [K^+^]_o_) (Fig. 2a) or hypokalaemic (4 or 3 mm [K^+^]_o_) (Fig. 2b,c) physiological buffer solutions, at a BCL of 125 ms. Reductions in [K^+^]_o_ to 4 and 3 mm did not significantly alter the endocardial APD_50_ (*n* = 18) (Table 2). However, these reductions in [K^+^]_o_ to 4 and 3 mm led to increases in epicardial APD_50_ values from 7.8 ± 0.8 to 16.9 ± 3.6 and 19.5 ± 2.4 ms respectively (*n* = 18) (Table 1). Reduction of [K^+^]_o_ from 5.2 to 4 mm led to significant increases in mean *epicardial* APD_70_ and APD_90_ values, from 19.7 ± 2.3 to 31.4 ± 4.9 ms and from 37.2 ± 1.7 to 58.4 ± 4.1 ms respectively (*P* < 0.05) (*n* = 7) (Table 1, Fig. 3a,b: clear columns). *Endocardial* MAP values were also affected. Admittedly mean endocardial APD_70_ values were not significantly affected by this initial reduction in [K^+^]_o_ from 5.2 to 4 mm (*n* = 7) (Table 2). Mean endocardial APD_90_ values were, however, significantly increased from 51.6 ± 1.9 to 62.8 ± 2.8 ms (*P* < 0.05) (*n* = 7) (Table 2, Fig. 3a,b: grey columns). These effects led to a marked reduction in both TDR and ΔAPD_90_ from 14.4 ± 2.6 ms under normokalemic conditions of 5.2 mm [K^+^]_o_, to 4.4 ± 5.0 ms upon lowering [K^+^]_o_ to 4 mm [K^+^]_o_, that was attributable to a greater lengthening of the epicardial MAP over the endocardial MAP (Fig. 3a,b: black columns).

Further reductions in [K^+^]_o_ from 4 to 3 mm similarly led to further prolongation of mean epicardial APD_70_ and APD_90_ values to 37.2 ± 2.9 and 66.7 ± 2.1 ms respectively (*n* = 11) (Table 2, Fig. 3b,c: clear columns). A reduction from 4 to 3 mm [K^+^]_o_ led to no further significant change in endocardial APD_70_ and APD_90_ values (*n* = 11) (Table 1, Fig. 3b,c: grey columns). The preferential lengthening of epicardial APD_90_ values over endocardial APD_90_ values in isolated mouse hearts perfused with 3 mm [K^+^]_o_ led to a further reduction in TDR to 3.4 ms, and actually a negative ΔAPD_90_ value of −3.4 ± 6.0 ms, reflecting the greater epicardial compared with endocardial APD_90_ (Fig. 2d, Fig. 3c: black column).

### Hypokalaemia induces repolarization abnormalities in spontaneously beating hearts

**Figure 4:**
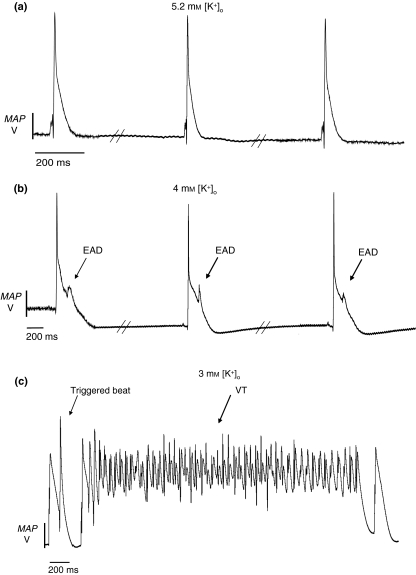
Representative left ventricular intrinsic epicardial MAP recordings from isolated, WT Langendorff-perfused mouse hearts under control conditions (a), and following perfusion with 4 mm [K^+^]_o_, (b) and 3 mm [K^+^]_o_ and (c) hypokalaemic solutions. Perfusion of hearts with 4 mm [K^+^]_o_ lead to the induction of EADs in three of seven preparations. Following perfusion with 3 mm [K^+^]_o_ buffer, EADs and triggered beats preceded periods of spontaneous, non-sustained VT in nine of 11 preparations.

Bradycardia is a known risk factor for TdP (Roden & Hoffman 1985), and earlier studies have reported EADs and TdP in rabbit hearts following the perfusion of a range of drugs implicated in acquired LQTS under combined states of hypokalaemia and bradycardia (Eckardt *et al.* 1998, Milberg *et al.* 2002). Accordingly, following the standard pacing protocols to accurately measure APD at various stages of repolarization, extrinsic pacing was terminated in all preparations, leading to a pronounced decrease in heart rate. Under these conditions, no repolarization abnormalities were ever recorded following perfusion with a normokalemic physiological solution. Control epicardial intrinsic MAPs displayed a typical triangular morphology, with a smooth repolarization phase (Fig. 4a). However, following reductions in [K^+^]_o_ to 4 mm, under intrinsic pacing conditions, EADs were now recorded from three of seven hearts (Fig. 4b). In these unprovoked preparations, EADs presented as pronounced positive deflections occurring in the smooth repolarization phase of the AP. Further reductions in [K^+^]_o_ to 3 mm frequently lead to salvos of triggered beats that preceded periods of non-sustained VT (VT) in nine of 11 preparations (Fig. 4c).

### PES induces ventricular tachycardia in hypokalaemic mouse hearts

The final experiments related the phenomena characterized above to an actual generation of arrhythmogenesis following a PES procedure. PES was used as an experimental tool to determine the arrhythmic susceptibility of isolated WT mouse hearts perfused with hypokalaemic (4 or 3 mm [K^+^]_o_) physiological buffer solutions. The PES procedures were directly adapted from clinical diagnostic techniques used to assess arrhythmogenic propensity in patients, for the current murine whole-heart model (Saumarez & Grace 2000, Balasubramaniam *et al.* 2003).

**Figure 5:**
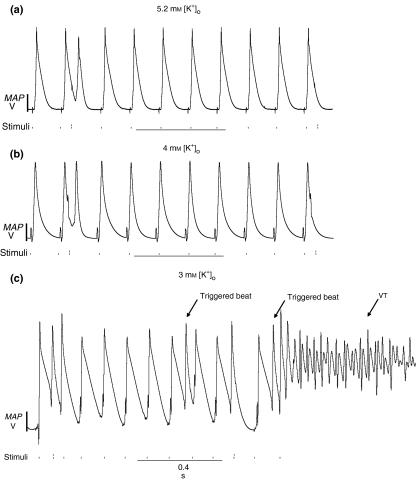
Programmed electrical stimulation (PES) of isolated, WT Langendorff-perfused mouse hearts under control conditions (a) and following perfusion with 4 mm [K^+^]_o_ (b) and 3 mm [K^+^]_o_ (c) hypokalaemic buffer solutions. PES repeatedly failed to induce VT in any preparation perfused with control, normokalaemic buffer. PES lead to the induction of VT in two of seven hearts perfused with 4 mm [K^+^]_o_ (shown is an example trace of one of the five hearts perfused with 4 mm [K^+^]_o_ in which PES failed to induce VT). Hearts perfused with 3 mm [K^+^]_o_ showed a high incidence of VT following PES (nine of 11 preparations).

Short S1–S2 coupling intervals under normokalemic (5.2 mm [K^+^]_o_) baseline conditions elicited typical extrasystolic APs (Fig. 5a). Figure 5 illustrates epicardial MAP recordings from isolated, perfused WT mouse hearts subjected to PES following progressive reductions in [K^+^]_o_. PES repeatedly failed to induce VT in isolated WT hearts under normokalemic (5.2 mm [K^+^]_o_) baseline conditions (*n* = 7) (Fig. 5a). Reducing [K^+^]_o_ from 5.2 to 4 mm led to the induction of VT in only two of seven preparations subjected to PES (29% incidence), in close parallel with clinical case reports of cardiac arrhythmias from hypokalaemic patients (Cohen *et al*. 1987). Figure 5b, illustrates a heart in which was not induced following perfusion with 4 mm [K^+^]_o_). Upon further reduction of [K^+^]_o_ to 3 mm, triggered beats and non-sustained VT in nine of 11 preparations were seen during PES protocols (Fig. 5c).

### Patch-clamp study of the effects of hypokalaemia on transient outward and inward potassium currents from epicardial and endocardial cardiac myocytes

To compliment the whole-heart electrophysiological findings, the experiments proceeded to explore the effects of hypokalaemia at the single-cell level. Individual myocytes were selectively isolated from the left ventricular epicardial and endocardial surfaces as described in Methods. The whole-cell configuration of the patch-clamp technique was used to record repolarizing K^+^ channel currents in epicardial and endocardial myocytes in normokalemic and hypokalaemic physiological buffer solutions.

**Figure 6:**
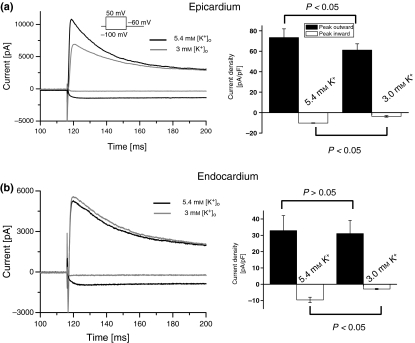
Outward and inward K^+^ currents recorded from epicardial and endocardial myocytes in normokalemic (black lines) and 3 mm [K^+^]_o_ hypokalaemic (grey lines) solutions using the whole-cell configuration of the patch-clamp technique. Under control conditions epicardial myocytes (a) exhibited a significantly greater early outward K+ current component compared with endocardial myocytes (b). Hypokalaemia significantly reduced early outward K^+^ current in epicardial cells (*n* = 4) (a) but had no such effects in endocardial cells (*n* = 5) (b). Hypokalaemia significantly reduced inward *I*_K1_ by equal extents in epicardial (*n* = 4) (a) and endocardial (*n* = 5) (b) myocytes.

To record a transient outward current (*I*_to_), cells were voltage-clamped at −60 mV and depolarized to 50 mV for a 500-ms duration. Under normokalemic conditions, average amplitude of *I*_to_ as reflected in the early peak of the outward current, in epicardial cells was significantly greater than in endocardial cells (73.46 ± 8.45 and 32.87 ± 9.27 pA/pF, respectively, *P* < 0.05, *n* = 9) (Fig. 6a,b respectively). We additionally applied hyperpolarizing steps from a holding potential of −60 to −100 mV to record an inwardly rectifying K^+^ channel current (*I*_K1_). Mean *I*_K1_ density was not significantly different between epicardial and endocardial cells (−10.18 ± 0.28 vs. −9.62 ± 1.65 pA/pF, respectively, *P* > 0.05, *n* = 9) (Fig. 6a,b).

Under hypokalaemic conditions of 3 mm [K^+^]_o_, *I*_to_ density was significantly reduced to 61.16 ± 6.14 pA/pF in epicardial cells (*P* < 0.05, *n* = 4) (Fig. 6a). However, *I*_to_ density was not significantly affected in endocardial cells under identical hypokalaemic conditions (32.87 ±9.27 and 31.09 ± 8.03 pA/pF, respectively, *P* > 0.05, *n* = 5) (Fig. 6b). Under hypokalaemic conditions *I*_K1_ density was significantly reduced from −10.18 ± 0.28 to −3.66 ± 0.77 pA/pF in epicardial cells (*P* < 0.05, *n* = 4) (Fig. 6a). Similarly, hypokalaemia significantly reduced *I*_K1_ in endocardial cells from −9.62 ± 1.65 to −2.93 ± 0.35 pA/pF (*P* < 0.05, *n* = 4) (Fig. 6b). However, reduction in *I*_K1_ under hypokalaemic conditions in epicardial cells was not significantly different from endocardial cells (−3.66 ± 0.77 vs. 2.93 ± 0.35 pA/pF; *P* > 0.05).

## Discussion

Clinical findings suggest that hypokalaemia may have intrinsic arrhythmogenic effects but the underlying physiological mechanisms remain unclear. We accordingly sought to investigate the intrinsic arrhythmogenic effects of hypokalaemia in isolated, Langendorff-perfused wild-type (WT) mouse hearts for the first time by recording MAPs from endocardial and epicardial left ventricular sites. The mouse model has proven to offer a powerful tool for the study of arrhythmias and their associated risk factors in murine hearts harbouring specific cardiac ion channel mutations that are known to directly correspond to human LQTS subtypes (Papadatos *et al.* 2002, Balasubramaniam *et al.* 2003). This study represents for the first time a quantitative description of the effects of hypokalaemia upon the occurrence of early EADs, VT and transmural changes in APD at the whole-heart and single-cell level. The results fully recapitulate clinical case reports of VT and TdP documented in hypokalaemic patients (Berthet *et al.* 1999, Kusano *et al.* 2001).

The present studies using Langendorff-perfused mouse hearts led to several new important conclusions. Firstly, we have confirmed for this isolated, perfused murine whole-heart model that under control conditions, endocardial MAPs were reproducibly longer in duration than epicardial MAPs. Our results confirm previous AP recordings from isolated murine myocytes (Guo *et al.* 1999), and from murine whole-heart preparations (Anumonwo *et al.* 2001, Casimiro *et al.* 2001, Knollmann *et al.* 2001).

In the present study, the greater APD of endocardial over epicardial MAPs led to a transmural APD gradient of 14.4 ± 2.6 ms, which closely correlates with a previous study of *in vivo* murine MAP recordings (Liu *et al.* 2004). The transmural difference in APD across the ventricular wall is important in establishing normal TDR-refractoriness, which may help to prevent re-entrant arrhythmias. Alterations in the normal patterns of cardiac repolarization and refractoriness are known contributing factors to re-entrant arrhythmias (Janse & Wit 1989).

Secondly, it was successfully shown that a reduction in [K^+^]_o_ leads to marked prolongation of epicardial and endocardial ventricular MAPs in *mouse* hearts, and a subsequent reduction in the transmural gradient of APD and therefore the TDR. To our knowledge this observation has not been reported on earlier occasions. Although a reduction in TDR is generally considered to reduce rather than increase the likelihood of arrhythmogenesis, one potential outcome of altered myocardial repolarization gradients would be an increased probability of repolarization gradient collision. This in turn would facilitate the generation of local conduction block and of consequent re-entrant arrhythmogenesis (Wolk *et al.* 1999, Rithalia *et al.* 2001). This could increase the susceptibility of the heart to arrhythmias initially induced by premature ventricular excitation through physiological phenomena such as EADs and triggered beats or via artificial premature excitation using PES. This first report of such phenomena in the isolated, perfused whole-heart model complements one previous study in isolated endocardial and epicardial rat myocytes that suggested that a reduction in TDR might also be proarrhythmic (Rithalia *et al.* 2001). Our results in the intact myocardium directly demonstrate that a reduced transmural gradient in APD can afford a mechanism of proarrhythmia.

Thirdly, we have established that the reduced heart rate seen in spontaneously beating hypokalaemic hearts led to an increased propensity for the development of repolarization abnormalities, such as EADs and triggered beats, which preceded episodes of VT. Although reduction of outward K^+^ currents observed under hypokalaemic conditions cannot *directly* initiate arrhythmogenic mechanisms such as EADs, AP prolongation will ensue, which is a known arrhythmogenic mechanism of action (Clancy *et al.* 2003). Prolongation of repolarization through reductions in repolarizing K^+^ currents has been speculated to induce EADs through Ca^2+^ channel reactivation (Haverkamp *et al.* 2000). Increases in the time spent in the voltage window range for L-type Ca^2+^ channel reactivation through AP prolongation are likely to generate EADs, which may in turn give rise to salvos of premature potentials termed triggered beats (January *et al.* 2000, Fabritz *et al.* 2003). Bouchard *et al.* (2004) demonstrated that prolonged exposure of rabbit ventricular myocytes to hypokalaemic solutions led to fluctuations in membrane potential and subsequent oscillations in cell length. These oscillations were shown to be because of Ca^2+^ entry through L-type Ca^2+^ channels.

Intrinsically beating hearts perfused with a reduced [K^+^]_o_ buffer of 4 mm, elicited EADs in three of seven preparations. Further reductions in [K^+^]_o_ to 3 mm, elicited not only EADs but also triggered beats that were followed by episodes of non-sustained VT in nine of 11 preparations. These findings at the level of the intact heart correlate with earlier cellular studies. Such studies have shown that the occurrence of EADs is increased under low frequency pacing, implicating the L-type Ca^2+^ current as a necessary depolarizing charge carrier during the EAD which appears to predominate under slow stimulation rates (Damiano & Rosen 1984, Zeng & Rudy 1995).

Reduction of [K^+^]_o_ to 3 mm therefore leads to a situation that contains high levels of EADs and triggered beats and the presence of both a further reduced TDR and a negative ΔAPD_90_ value, representing the first point at which epicardial exceeds endocardial APD_90_. This would be expected to lead to a proarrhythmic state, in which there is on the one hand an increased likelihood of EADs, and on the other in which an EAD is likely to give rise to development of VT only in the setting of markedly altered transmural gradients in APD. In this situation, induction of VT is considered to result from *both* a trigger and an appropriate substrate.

Finally, it was demonstrated that the occurrence of VT using PES correlated with reductions in both TDR and ΔAPD_90_, concomitant with a progressive reduction in [K^+^]_o_. The present results document that a reduction in [K^+^]_o_ to 4 mm leads to a 29% incidence of VT, closely paralleling the clinical case study of Cohen *et al.* (1987), who reported a similar frequency of VT amongst hypertensive patients receiving diuretic therapy. This finding further validates the use of the intact, isolated, Langendorff-perfused mouse heart as an experimental set-up to accurately study human arrhythmogenecity. Following further reduction in [K^+^]_o_ to 3 mm, PES induced VT in nine of 11 preparations.

This study of arrhythmogenesis in the intact mouse heart complements previous studies on the molecular effects of hypokalaemia at the cellular level. Reductions in [K^+^]_o_ have been shown to reduce the conductances of a number of K^+^ channels including the transient outward current (*I*_to_) (Firek & Giles 1995), the rapidly activating delayed rectifier current (*I*_Kr_) (Scamps & Carmeliet 1989, Yang & Roden 1996) and the inwardly rectifying current (*I*_K1_) (Carmeliet 1982, Bouchard *et al.* 2004). Outward potassium current in response to depolarization (−60 to 50 mV) and inward rectifying current (*I*_K1_) in response to hyperpolarization (−60 to −100 mV) were measured in normokalemic (5.4 mm) and hypokalamic (3 mm) buffer from both epicardial and endocardial myocytes.

Firstly, under normokalemic conditions we recorded a greater earlier outward K^+^ current, attributable to a transient outward (*I*_to_) current component, from epicardial compared with endocardial myocytes. Mouse cardiac repolarization is dominated by the rapidly activating *I*_to_ K^+^ current (Nerbonne *et al.* 2001). *I*_to_ is differentially expressed in the murine ventricle, with higher protein levels found in the epicardium than the endocardium (Brunet *et al.* 2004). Such differences in the transmural expression of *I*_to_ are thought to account for the shorter APDs frequently reported at the murine ventricular epicardium compared with the endocardium (Knollmann *et al.* 2001). In the present study, this result compliments our MAP recordings from isolated perfused hearts, in which we demonstrated that APs recorded from the endocardial surface were greater in duration than APs recorded from the epicardial surface. Thus, the difference in *I*_to_ density between the epicardium and the endocardium may help explain why APs recorded from these two sites significantly differ in duration.

Secondly, under hypokalaemic conditions, we recorded a significant reduction of early outward current, attributable *I*_to_, in epicardial myocytes. Firek & Giles (1995) reported that reductions in [K^+^]_o_ reduced *I*_to_ in human atrial myocytes. Reductions in *I*_to_ can prolong APD and increase Ca^2+^ entry via L-type Ca^2+^ channels (Fiset & Giles 2006). However, under similar conditions, early outward current was not significantly affected in the endocardial myocytes. Reductions in epicardial but not endocardial *I*_to_ under hypokalaemic conditions correlates with preferential lengthening of epicardial compared with endocardial APD under hypokalaemic conditions reported in the present study at the whole-heart level. Furthermore, we documented a significant increase in the early repolarization phase, as reflected in epicardial APD_50_, at the whole-heart level under hypokalaemic conditions of 3 mm [K^+^]_o_, supporting the notion that an early outward K^+^ current, most likely *I*_to_, is reduced in epicardial myocytes under hypokalaemic conditions. Thus, a reduction in *epicardial I*_to_ under hypokalaemic conditions could account for preferential epicardial APD prolongation at 50%, 70% and 90% repolarization and could therefore be considered one of the primary mechanisms responsible for the change in the transmural gradient of repolarization, reflected by changes in ΔAPD_90_ seen at the whole-heart level.

Thirdly, we also recorded K^+^ current through *I*_K1_ by applying hyperpolarizing steps. Under normokalemic conditions, inward *I*_K1_ was not significantly different between epicardial and endocardial myocytes. Under hypokalaemic conditions, inward *I*_K1_ current was significantly reduced by equal extents in both epicardial and endocardial myocytes. Therefore, it is unlikely that a differential reduction in *I*_K1_ between epicardial and endocardial myocytes may significantly contribute to an altered transmural gradient of repolarization.

Hypokalaemia induces hyperpolarization of the cell membrane, which inhibits *I*_K1_ (Carmeliet 1982, Bouchard *et al.* 2004). *I*_K1_ is the main current responsible for setting the resting membrane potential in mammalian heart cells and it can also contribute to the late phase of repolarization (Nichols *et al.* 1996). Inhibition of outward-going *I*_K1_ via hypokalaemia-induced hyperpolarization of the cardiac cell membrane would therefore be expected to prolong the later phases of cardiac AP repolarization. Such effects could account for the significant epicardial and endocardial AP prolongation at 90% repolarization in isolated hearts perfused with 3 mm [K^+^]_o_. Previously, genetically engineered mice lacking *I*_K1_ exhibit cardiac AP prolongation (Zaritsky *et al.*, 2001). However, we understand that this is not a definitive experimental approach to asses the effect of *I*_K1_ in AP repolarization under hypokalaemic conditions as the physiological function of *I*_K1_ is because of a smaller outwardly rectifying component of *I*_K1_ current. Under the conditions of our patch-clamp experiments, however, a large early transient outward K^+^ current will mask any outward *I*_K1_ current.

The results from the whole-heart and single-cell electrophysiological studies strongly suggest that reductions in an early outward K^+^ current, most likely to be *I*_to_, is the primary ionic mechanism for the significant increase in epicardial APD at 50%, 70% and 90% repolarization and for the alteration in the transmural gradients of repolarization observed under hypokalaemic conditions. Previously, *I*_Kr_ has been shown to be similarly sensitive to reductions in [K^+^]_o_ through either increased channel inactivation kinetics (Yang *et al.* 1997) or through an increased inhibitory effect of Na^+^ ions at an extracellular binding site of the human *ether-a-go-go related gene* (HERG) K^+^ channel, which constitutes *I*_Kr_, as [K^+^]_o_ is lowered (Numaguchi *et al.* 2000). HERG K^+^ channels rapidly activate from closed to open states during depolarization, but pass little outward current as they rapidly inactivate (Vandenberg *et al.* 2001). Channels subsequently pass an outward current as they recover from inactivation during repolarization (Clancy *et al.* 2003). Thus the corresponding murine HERG K^+^ channel (mERG) may contribute to the late phase of murine repolarization and reductions in *I*_Kr_ may be responsible for increased epicardial and endocardial APD_90_ observed at the whole-heart level under hypokalaemic conditions. When recording outward K^+^ currents at the single-cell level in hypokalaemic solutions, we recorded a significant reduction of outward current at early times in epicardial myocytes, more likely reflecting *I*_to_ as opposed to *I*_Kr_. Nevertheless we have taken the care to emphasize early outward K^+^ current as opposed to individual outward K^+^ current components. At the whole-heart level we report significant epicardial AP prolongation at 3 mm [K^+^]_o_ occurring at early repolarization times, as reflected by increased APD_50_, consistent with the single-cell findings and further supporting the notion that an early outward K^+^ current, attributable to *I*_to_, is reduced in the epicardium under hypokalaemic conditions.

However, this does not exclude the possibility of other mechanisms contributing to arrhythmogenesis under hypokalaemic conditions. Reductions in [K^+^]_o_ have been associated with electrogenic Na^+^/K^+^ ATPase pump inhibition (Eisner & Lederer 1979) and a subsequent increase in [Na^+^]_i_ (Boyett *et al.* 1986). Elevations in [Na^+^]_i_ may lead to an increase in [Ca^2+^]_i_ through inhibition of Ca^2+^ extrusion via the Na^+^–Ca^2+^ exchanger (White & Terrar 1991). Nevertheless, the isolated, perfused heart electrophysiological data alongside the single-cell patch-clamp data in the present study strongly supports the notion that the reduction of early repolarizing K^+^ currents selectively in the epicardium leading to AP prolongation and the subsequent induction of EADs, alongside alteration of transmural gradients of repolarization is the primary arrhythmogenic mechanism of action associated with hypokalaemia in the mouse heart.

In conclusion, analysis of epicardial and endocardial MAPs recorded from isolated, Langendorff-perfused, WT murine whole-heart preparations and patch-clamp K^+^ current measurements in isolated epicardial and endocardial myocytes under varying degrees of hypokalaemia has thus shed new light on the pathogenesis of VT under hypokalaemic conditions. Here we report for the first time episodes of EADs, triggered beats and VT in the setting of a reduced TDR recorded from intact, isolated mouse hearts perfused with hypokalaemic solutions, further highlighting the possible severe clinical consequences of relatively small reductions in serum potassium levels. Furthermore, at the single-cell level we report a significant reduction in early outward current in epicardial myocytes under hypokalaemic conditions, an effect that is likely to play an important role in the generation of altered transmural gradients of repolarization seen at the whole-heart level. These data suggest that treatment of even modest hypokalaemia is critical in preventing serious unwanted lethal cardiac events. Intervention of serum [K^+^] may prove to be beneficial in the prophylaxis of VT and TdP induced by hypokalaemia.
